# The Third Finger Middle Phalanx Maturation (MPM) Method to Assess Timing of Functional Treatment for Skeletal Class II Malocclusion: Report of Three Cases

**DOI:** 10.1155/2019/8382612

**Published:** 2019-07-24

**Authors:** Giuseppe Perinetti

**Affiliations:** Private practice, Nocciano (PE), Italy

## Abstract

A deficient mandibular growth on the sagittal plane is the most frequent diagnostic finding in dentoskeletal Class II malocclusion. Evidence indicated that functional treatment for such malocclusion is efficient only if performed during the pubertal growth spurt, as identified through radiographical growth indicators. With the aim of reducing the radiation to the patients and to follow longitudinally individual growth phases, the use of the sole third finger middle phalanx maturation (MPM), as a 5-stage method, has been proposed. Herein, three clinical cases of skeletal Class II malocclusion in growing patients treated by removable functional appliances (with or without full-fixed appliance treatment) are reported. Timing of intervention was strictly planned according to the MPM method, and skeletal effects have been recorded up to 21 months of follow-up. In all the cases, noteworthy skeletal effects have been achieved in terms of mandibular elongation, with relevant occlusal and aesthetic outcomes. It has also been showed that results are stable or slightly improved after functional treatment. These results would be achieved irrespective of the appliance used and support the use of the MPM method in everyday clinical practice.

## 1. Introduction

A deficient mandibular growth on the sagittal plane is the most frequent diagnostic finding in dentoskeletal Class II malocclusion that occurs in up to one-third of the population [1]. In spite of the relevant literature and the wide use of functional treatment for skeletal Class II malocclusion, its efficiency is still controversial [2] with reviews reporting very limited [3], partial [4], or relevant [5] skeletal effects in terms of induced mandibular growth. While some types of malocclusion are well known to be treated successfully at an early stage of development [6, 7], some clinical trials indicated that functional treatment for skeletal Class II malocclusion is the most efficient when performed during the pubertal growth spurt (for review, see [2]). In this context, efforts have been carried out to find reliable and reproducible indicators of the onset of pubertal growth spurt, i.e., skeletal maturation, in individual subjects [2]. These indicators include the hand-and-wrist maturation (HWM) [8] and cervical vertebral maturation (CVM) [9] methods that have been used extensively as stage-based methods. However, irrespective of the radiographical indicator, these stages have variable duration [8, 10, 11], making the precise identification of the timing of intervention reliable only if a longitudinal monitoring of the passage to one stage to the following one (also referred to as ossification event [12]) is followed. Therefore, patients should ideally be followed from a prepubertal stage of development to the pubertal one. In this context, a crucial issue relates to the clinical feasibility of the repetition of the recording by invasive X-ray procedures [13, 14], irrespective of whether the used growth indicator is accurate, thus limiting the use of indicators such as the HWM and CVM methods.

**Figure 1:**
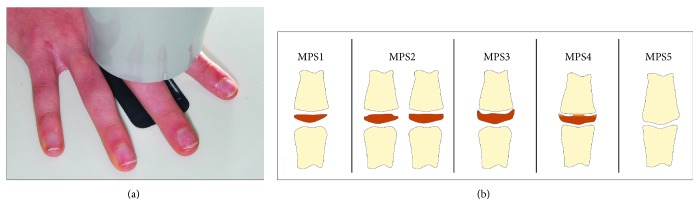
(a) Recording of the third finger middle phalanx film through an intraoral X-ray unit. (b) The stages of the third finger middle phalanx maturation (MPM) method (reposted as MPS, see text for details).

With the aim of reducing the radiation to the patients, the use of the sole third finger middle phalanx for a maturational method has been proposed [15, 16]. Therefore, this middle phalanx maturation (MPM) method [11] may constitute a valid compromise between the necessity of longitudinal recordings and acceptable radiation exposure to the patient, and its use can be indicated for planning timing of functional treatment for skeletal Class II malocclusion [2, 11]. In spite of the potential clinical advantages offered by the MPM method, clinical applications still have to be reported, especially when dealing with growing skeletal Class II patients that needs to be treated functionally. This method has advantages such as absence of double contours or superimposition by other structures and it would be of easy execution, as it may be performed in any clinical setting, through intraoral X-ray units and periapical films (using setting for mandibular incisors). In the improved version of the 5-stage MPM method recently reported [11], MPM stage 2 has been reported to precede the mandibular growth peak, which is generally concomitant to the subsequent stage 3, with an overall diagnostic accuracy of 0.91. MPM stages 2 and 3 have been considered associated with the onset and maximum mandibular growth peak, respectively. Details of each stage are as follows (Figure 1): MPM stage 1, attained before the onset of the mandibular growth peak: epiphysis is narrower than the metaphysis, or epiphysis is as wide as metaphysis but with both tapered and rounded lateral borders. Epiphysis and metaphysis are not fused; MPM stage 2, attained at coincidence with the onset of the mandibular growth peak: epiphysis is at least as wide as the metaphysis with sides increasing thickness and showing a clear line of demarcation at right angle, either with or without lateral steps on the upper contour; MPM stage 3, attained at coincidence of the maximum mandibular growth peak: epiphysis is either as wide as or wider than the metaphysis with lateral sides showing an initial capping towards the metaphysis. Epiphysis and metaphysis are not fused; MPM stage 4, attained after the mandibular growth peak: epiphysis begins to fuse with the metaphysis although contour of the former is still clearly recognizable; and MPM stage 5, when epiphysis is totally fused with the metaphysis. For all the stages, in case of asymmetry between the two sides, the more mature side is used to assign the stage.

To the best of my knowledge, only a single clinical case involving the MPM method has been reported [17]. However, this case represented a mild Class II malocclusion and lacked follow-up [17]. Similarly, previous clinical trials (using different indicators) were generally limited to the posttreatment effects lacking a follow-up [2] or were designed to include only prepubertal patients [18]. Herein, three clinical cases of skeletal Class II malocclusion in growing patients treated by removable functional appliances (with or without full-fixed appliance treatment) are reported. In each case, timing of intervention was strictly planned according to the MPM method and skeletal effects have been recorded up to 21 months of follow-up. An informed consent has been obtained from the parents of all three the patients.

## 2. Case Presentation

### 2.1. Case 1

**Figure 2:**
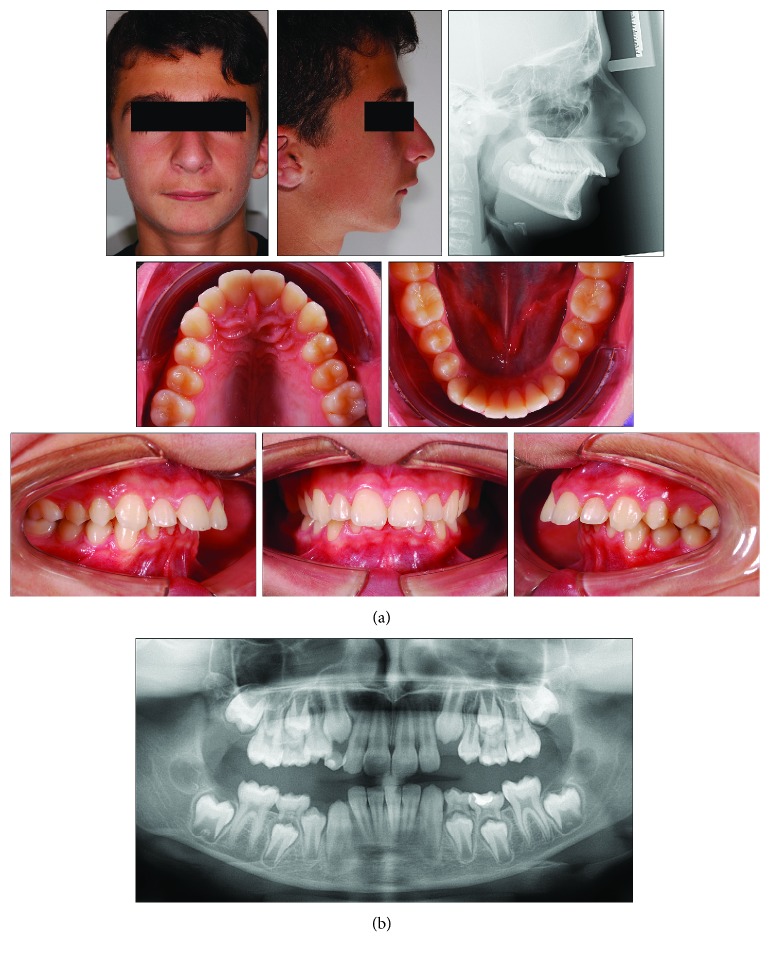
Case 1. (a) 9-year and 10-month-old male patient with skeletal Class II malocclusion, increased overjet, and overbite before treatment. (b) Panoramic radiograph taken at first visit when the patients was 9 years old.

**Figure 3:**
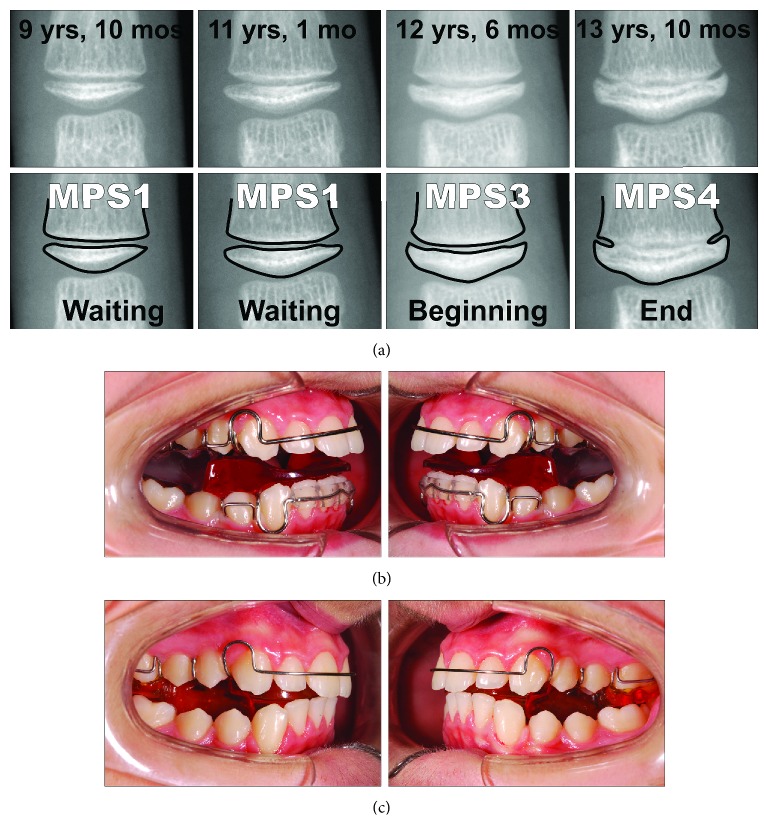
Case 1. (a) The series of the MPM recordings with corresponding ages and according to the timing of functional treatment (waiting before treatment, beginning of treatment, and end of treatment). (b) Intraoral views of the Twin-Block appliance. (c) Intraoral views of the removable Clark's retention appliance.

**Figure 4:**
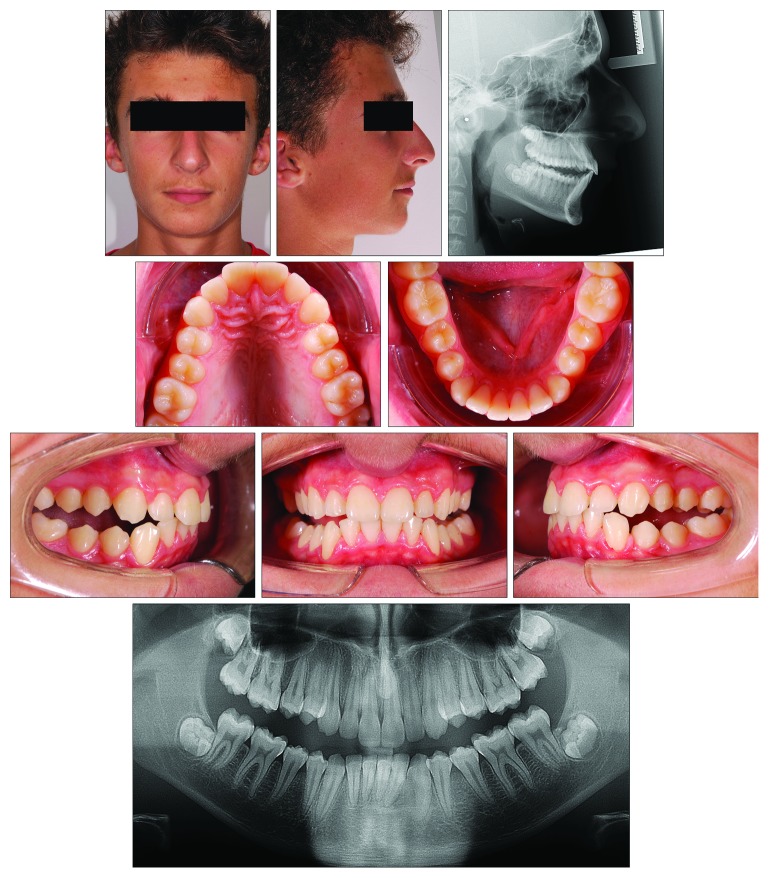
Case 1. Patient after 14 months of functional treatment with Twin-Block when he was 13 years and 10 months old.

A 9-year-old male presented with a clear dentoskeletal Class II malocclusion in late mixed dentition. Since a clinical and panoramic film analysis excluded any indication for an interceptive treatment, the patient was asked to present at yearly interval to monitor the skeletal maturation according to the MPM method (see below) until a pubertal growth stage is achieved. Treatment began when the patient was 12 years and 6 months old (Figure 2(a)) when he had a full permanent dentition with a bilateral full-cusp Class II molar relationship and noteworthy increased overjet (11.9 mm) and overbite. His medical history was not contributory. Soft tissue profile and cephalometric analysis suggested that Class II malocclusion (ANB, 6.3°; Wits appraisal, 10.5 mm) was mainly due to mandibular retrusion (SNB, 72.8°; Pog to Nasion perp., -12.6 mm) (Figure 2(a); Table 1). A hypodivergent growth pattern (SN to GoGn, 28.5°) was also seen with no major skeletal transverse maxillary deficiency (Figure 2(a); Table 1). A panoramic radiograph taken at 9 years revealed no significant anomalies (Figure 2(b)). The MPM staging was initially performed at 9 years and 10 months (stage 1) and again at 11 years and 1 month (stage 1) and 12 years and 6 months (stage 3) (Figure 3(a)). After this recording, functional treatment began by means of a Twin-Block appliance carrying the TheraMon® Chip (MC Technology GmbH, Hargelsberg, Austria) for patient compliance recording and a lower acrylic labial bow with the aim of preventing mandibular incisor proclination (Figure 3(b)). This Twin-Block appliance had an expansion screw (Model A4805-14R, Leone Orthodontic Products, Sesto Fiorentino (FI), Italy) in the upper block. Mandibular advancement for the bite construction was maximum with almost an edge-to-edge incisor relationship (Figure 3(b)). An expansion screw was activated once/twice per month (0.2/0.4 mm), and no modifications to the appliance were performed during treatment with the exception of the trimming of the upper block for lower molar extrusion, according to Clark's recommendations [19]. Patient cooperation was satisfactory with a mean wear time above 18 hours (not shown). Since parents of the patients refused further full-fixed appliance treatment, after 14 months of functional treatment, a removable Clark's retention appliance [19] was delivered for the retention of the results and to favor lateral open bite closing. Wearing was at night for 6 months (Figure 3(c)). After active functional treatment, the patient had a super Class I molar relationship along with a noteworthy lateral open bite on both sides as the side effect of the treatment [19]; second molars were in contact (Figure 4).

At the end of the functional treatment, the patient achieved MPM stage 4 (Figure 3(a)) and had a Class I dentoskeletal relationship (overjet reduced to 4.1 mm with 7.8 mm of improvement) with an ANB angle of 2.2° and a Wits appraisal of 1.6 mm (4.1° and 8.9 mm of improvement as compared to the pretreatment measurements, respectively) (Figure 4; Table 1). The Pog was advanced up to 5.4 mm. On the contrary, no relevant effects were seen in the maxilla, for which SNA, A to Nasion perp., and Palatal plane to FH plane underwent little or irrelevant changes. Over 16 months (14 of which of functional treatment), an increase in the total mandibular length as Co-Gn distance (including basal growth and growth induced by functional treatment) was equal to 7.9 mm. The panoramic radiograph revealed the presence of third molars in all quadrants and absence of any anomaly (Figure 4).

**Figure 5:**
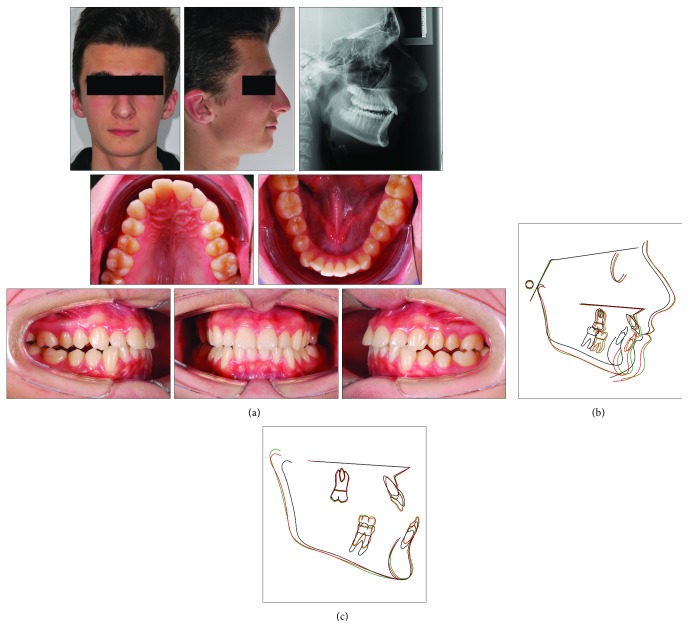
Case 1. (a) Patient after 18 months of follow-up (including 6 months of retention) when he was 15 years and 4 months old. (b) Superimposition of pretreatment (black), posttreatment (red), and follow-up (green) cephalometric tracings on stable structures of the anterior cranial base. (c) Regional superimposition of cephalometric tracings on the maxilla and mandible.

At the end of functional treatment, superimposition on the anterior cranial base [20] showed a notable forward displacement of the Pog and a slight clockwise rotation of the mandible (Figure 5(b)), with SN to GoGn increased by 1.5° (Table 1). The regional mandibular superimposition [20] demonstrated upward and backward growth of the condyle and ramus, along with extrusion of the molars and an irrelevant change in the incisor inclination (Figure 5(c); Table 1). The regional maxillary superimposition [20] demonstrated irrelevant first molar movements and a significant improvement of the incisor inclination over 10°, from 127.5° to 117.3° (Figure 5(c); Table 1).

Finally, stable results were seen at the 18-month follow-up (including 6 months of retention appliance wearing) in terms of an occlusion and skeletal relationship (Figure 5; Table 1). Although ANB angle and Wits appraisal remained stable with minimal changes, a further improvement of the mandibular retrusion was seen as Pog to Nasion perp. changed from -7.2 mm to -6.2 mm (Table 1). A slight lateral open bite was still present, while facial aesthetic dramatically changed, with resolution of the retruded mandible.

### 2.2. Case 2

**Figure 6:**
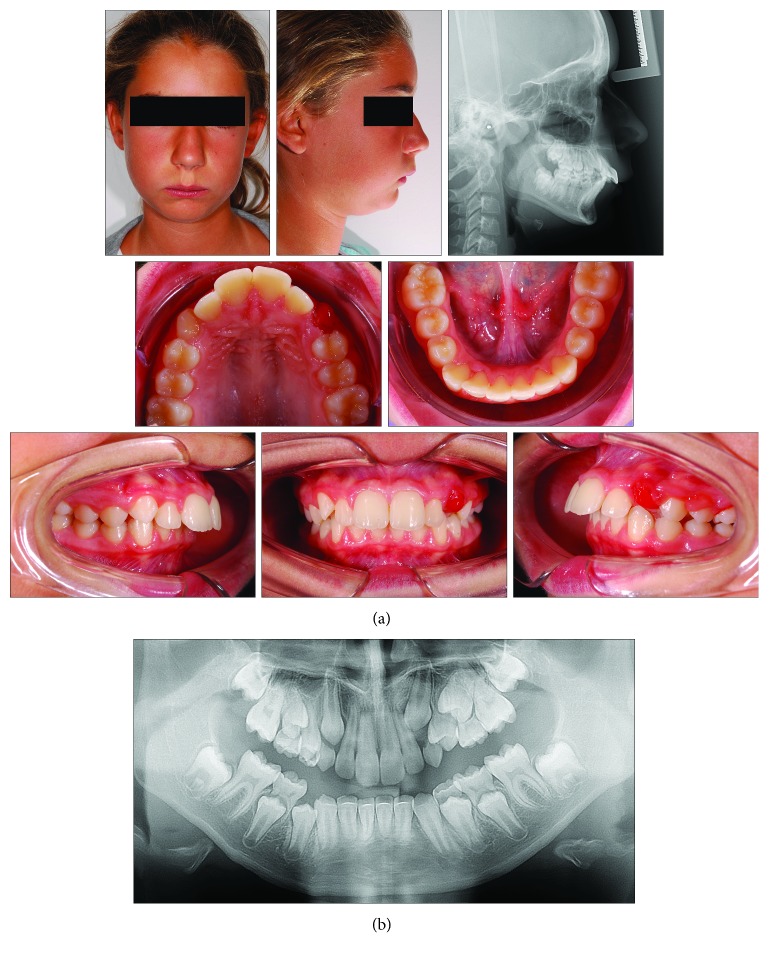
Case 2. (a) 9-year and 8-month-old female patient with skeletal Class II malocclusion, increased overjet, and overbite before treatment. (b) Panoramic radiograph taken at first visit when the patients was 8 years 10 months old.

**Figure 7:**
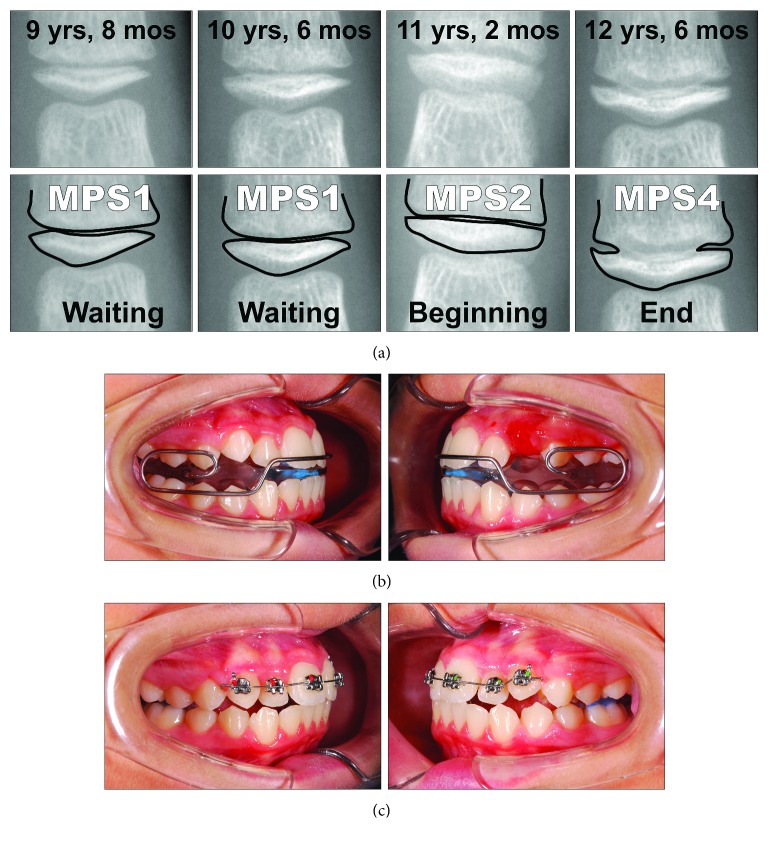
Case 2. (a) The series of the MPM recordings with corresponding ages and according to the timing of functional treatment (waiting before treatment, beginning of treatment, and end of treatment). (b) Intraoral views of the Bionator appliance. (c) The fixed appliance on the maxillary anterior teeth.

An 8-year and 10-month-old female presented with a clear dentoskeletal Class II malocclusion in late mixed dentition. Since a clinical and panoramic film analysis excluded any indication for an interceptive treatment, the patient was asked to present at yearly interval to monitor the skeletal maturation according to the MPM method (see below) until a pubertal growth stage is achieved. Treatment began when the patient was 11 years and 2 months old (Figure 6(a)), when she had an almost complete permanent dentition with a bilateral half-cusp Class II molar relationship and increased overjet (9.9 mm) and overbite. Her medical history was not contributory. Soft tissue profile and cephalometric analysis suggested that Class II malocclusion (ANB, 5.3°; Wits appraisal, 4.2 mm) was due to mandibular retrusion (SNB, 75.4°; Pog to Nasion perp., -6.5 mm) (Figure 6(a); Table 2). A normal vertical growth pattern (SN to GoGn, 30.1°) was also seen with no major skeletal transverse maxillary deficiency (Figure 6(a); Table 2). A panoramic radiograph taken at 8 years 10 months revealed no significant anomalies (Figure 6(b)). The MPM staging was initially performed at 9 years and 8 months (stage 1) and again at 10 years and 6 months (stage 1) and 11 years and 2 months (stage 2) (Figure 7(a)). After this recording, functional treatment began by means of a Bionator appliance carrying the TheraMon® Chip (Figure 7(b)). Mandibular advancement for the bite construction was maximum with an edge-to-edge incisor relationship (Figure 7(b)). Patient cooperation was satisfactory with a mean wear time above 16 hours (not shown). After 12 months of functional treatment, the patient had a super Class I molar relationship along with a crossbite of both the maxillary lateral incisors. Since parents of the patients refused a full-fixed appliance treatment, a 3-month long fixed treatment limited to the maxillary anterior teeth in combination with posterior occlusal pads was performed (Figure 7(c)). Subsequently, an upper Essix retainer was delivered to the patient who was instructed to wear at night.

**Figure 8:**
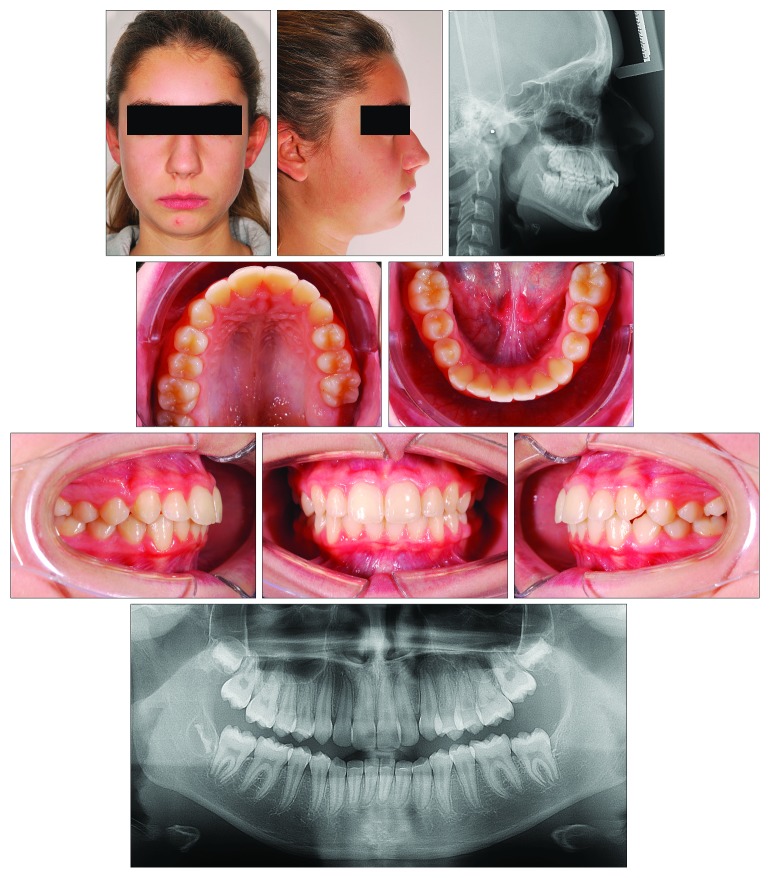
Case 2. Patient after 12 months of functional treatment with the Bionator and 3 months of fixed appliance treatment when she was 12 years and 6 months old.

At the end of the whole treatment that lasted 15 months, the patient achieved MPM stage 4 (Figure 7(a)) and had a Class I dentoskeletal relationship (overjet reduced to 4.9 mm with 5.0 mm of improvement) with an ANB angle of 3.3° and a Wits appraisal of -0.3 mm (2.0° and 4.5 mm of improvement as compared to the pretreatment measurements, respectively) (Figure 8; Table 2). The Pog was advanced to 1.7 mm. As for Case 1, no relevant effects were seen in the maxilla, for which SNA, A to Nasion perp., and Palatal plane to FH plane underwent little or irrelevant changes. Over 16 months (12 of which of functional treatment), an increase in the total mandibular length as Co-Gn distance (including basal growth and growth induced by functional treatment) was equal to 4.8 mm. The panoramic radiograph showed absence of any anomaly except for the agenesis of the mandibular left third molar.

**Figure 9:**
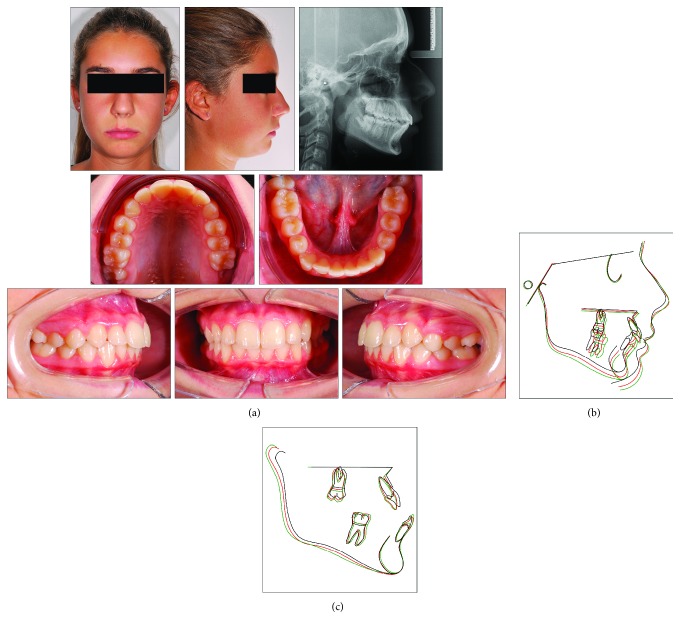
Case 2. (a) Patient after 16 months of follow-up when she was 13 years and 10 months old. (b) Superimposition of pretreatment (black), posttreatment (red), and follow-up (green) cephalometric tracings on stable structures of the anterior cranial base. (c) Regional superimposition of cephalometric tracings on the maxilla and mandible.

At the end of the whole treatment, superimposition on the anterior cranial base [20] showed a forward displacement of the Pog and a slight clockwise rotation of the mandible (Figure 9(b)), with SN to GoGn increased by 1.7° (Table 2). The regional mandibular superimposition [20] demonstrated upward and backward growth mainly in the condyle region, along with only a slight extrusion of the molars and lower incisor proclination (Figure 9(c); Table 2). The regional maxillary superimposition [20] demonstrated molar extrusion and a significant improvement of the incisor inclination by about 11°, from 124.5° to 115.6° (Figure 9(c); Table 2).

Finally, stable results were seen at the 16-month follow-up in terms of an occlusal and skeletal relationship (Figure 9; Table 2). As for Case 1, a further slight improvement of the skeletal relationship was seen during this follow-up term, as the ANB angle further decreased up to 0.4° (while Wits appraisal and Pog to Nasion perp. remained generally similar (Table 2)). Overall, the facial aesthetic improved noteworthily, with a correction of the convex profile.

### 2.3. Case 3

**Figure 10:**
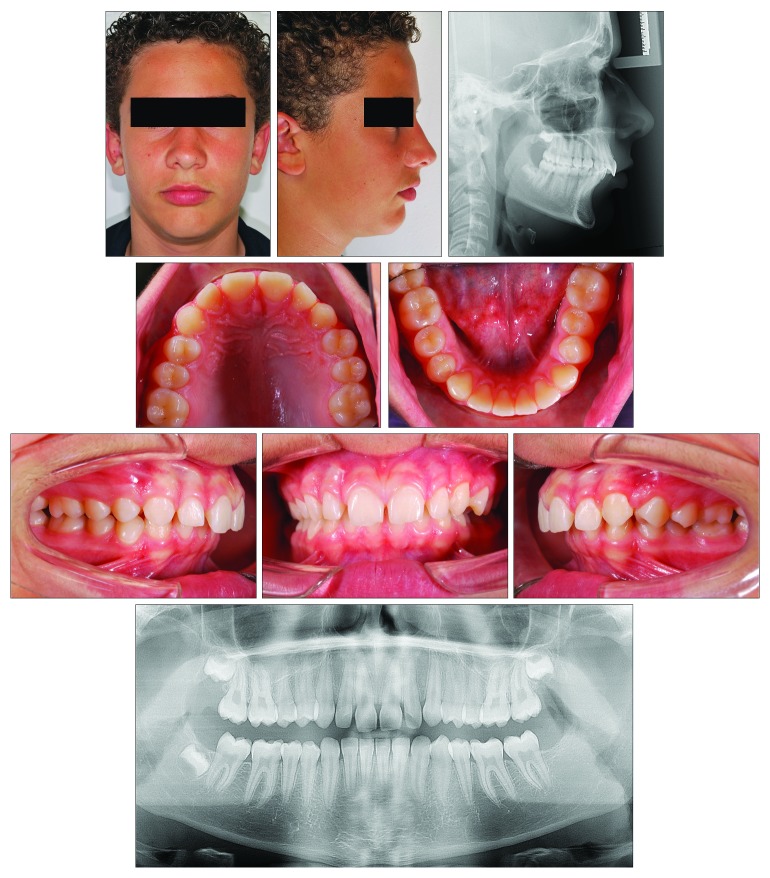
Case 3. 13-year-old male patient with skeletal Class II malocclusion, increased overjet, and overbite before treatment.

**Figure 11:**
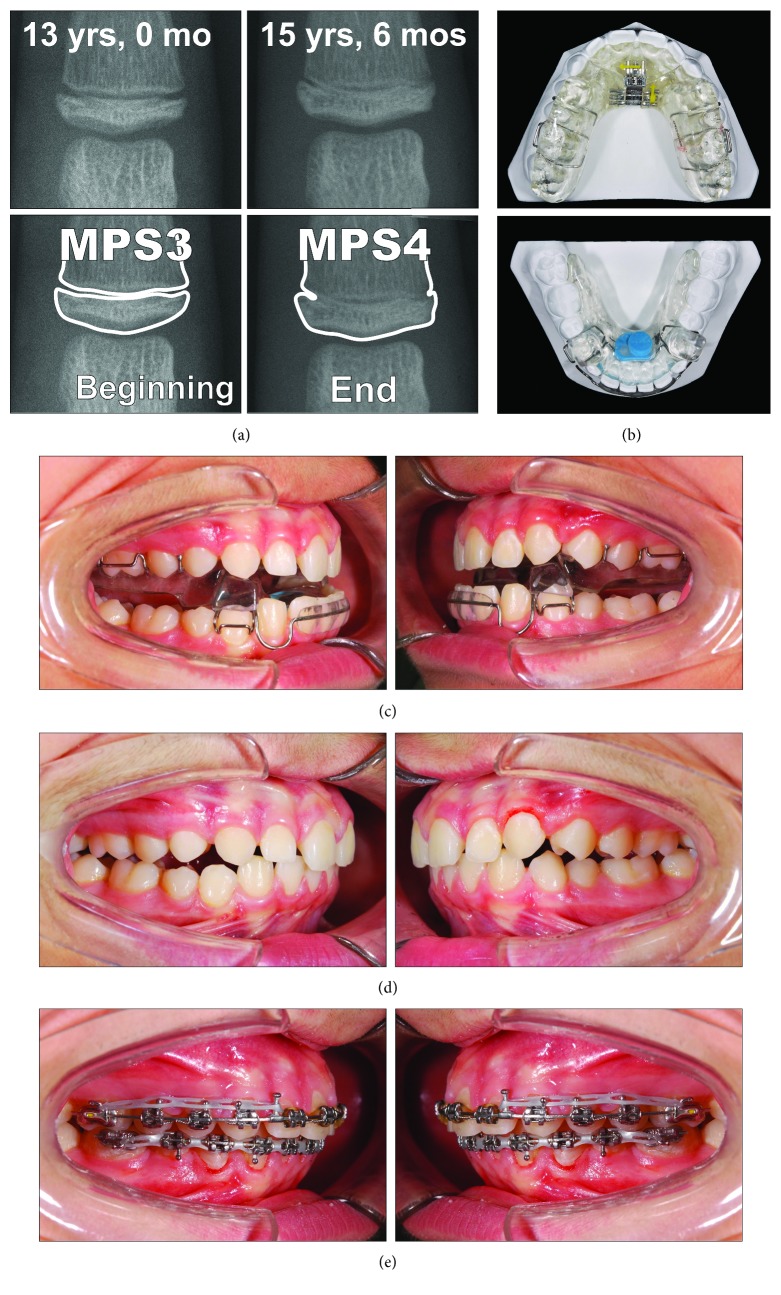
Case 3. (a) The MPM recordings with corresponding ages and according to the timing of functional treatment (beginning and end of treatment). (b) Occlusal views of the modified Twin-Block appliance. (c) Intraoral views of the modified Twin-Block appliance. (d) The full-fixed appliance.

A 13-year-old male (Figure 10) presented with a dentoskeletal Class II malocclusion, a bilateral half-cusp Class II molar relationship, and increased overjet and overbite, along with a scissor bite on the left side. His medical history was not contributory. As for the other cases, soft tissue profile and cephalometric analysis showed that Class II malocclusion (ANB, 7.1°; Wits appraisal, 7.2 mm) was mainly due to mandibular retrusion (SNB, 75.6°; Pog to Nasion perp., -8.6 mm) (Figure 10; Table 3). A normal vertical growth pattern (SN to GoGn, 32.2°) was also seen with no major skeletal transverse maxillary deficiency (Figure 10; Table 3). A panoramic radiograph revealed the agenesis of the left mandibular third molar and no other anomalies. At the moment, the patient presented he had MPM stage 3 (Figure 11(a)); therefore, functional treatment was started immediately by means of a modified Twin-Block appliance carrying the TheraMon® Chip. This modified Twin-Block appliance had a lower acrylic labial bow (as that in Case 1) and a three-way screw (Model A0930-14, Leone Orthodontic Products) in the upper block allowing a simultaneous increase in the transverse dimension and prevention of upper incisor reclination (Figure 11(b)). Mandibular advancement for the bite construction was maximum with an edge-to-edge incisor relationship (Figure 11(c)). No modifications to the appliance were performed during treatment with the exception of the trimming of the upper block for lower molar extrusion, according to Clark's recommendations [9]. A frontal screw was activated once/twice a month (0.1/0.2 mm). After 10 months of functional treatment with the Twin-Block, the patient had a super Class I molar relationship along with a bilateral open bite as the side effect of the treatment [19] (Figure 11(b)). Patient cooperation was satisfactory with a mean wear time above 18 hours (not shown). Immediately after the end of the Twin-Block treatment (Figure 11(d)), the patient underwent a second phase of fixed orthodontic treatment with an MBT straight-wire multibracket appliance (Optimus, Effedental, Barbeano di Spilimbergo (PN), Italy) to refine the occlusion (Figure 11(e)). This treatment involved the use of intermaxillary elastics and lower incisor stripping to reduce their inclination to the mandibular plane. At the end of this treatment, Essix retainers were delivered to the patient who was instructed to wear at night.

**Figure 12:**
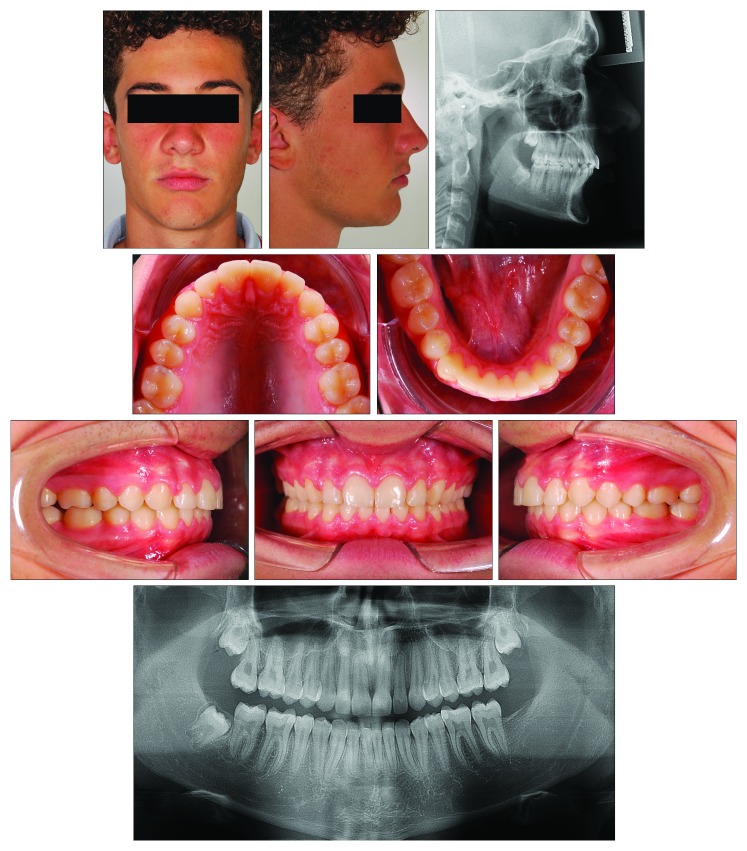
Case 3. Patient after 10 months of functional treatment with modified Twin-Block and 18 months of full-fixed appliance treatment when he was 15 years and 6 months old.

At the end of the full-fixed appliance treatment that lasted 18 months, the patient achieved an MPM stage 4 (Figure 11(a)) and had a Class I dentoskeletal relationship with an ANB angle of 3.9° and a Wits appraisal of 4.0 mm (3.2° and 3.2 mm of improvement as compared to the pretreatment measurements, respectively) (Figure 12; Table 3). The Pog was advanced up to 7.2 mm. As for the other cases, no relevant effects were seen in the maxilla, for which SNA, A to Nasion perp., and Palatal plane to FH plane, underwent little or irrelevant changes. Over 27 months (10 of which of functional treatment), an increase in the total mandibular length as Co-Gn distance (including basal growth and growth induced by functional treatment) was equal to 8.4 mm. The panoramic radiograph showed a good root parallelism.

**Figure 13:**
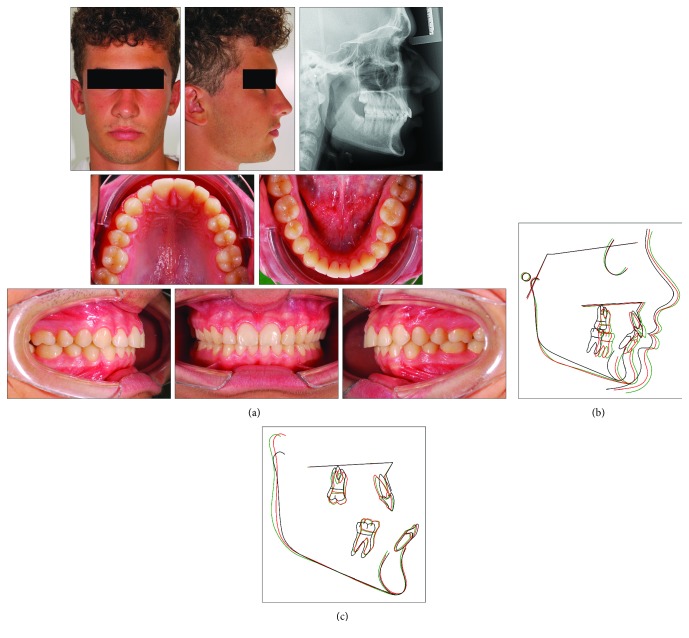
Case 3. (a) Patient after 21 months of follow-up when he was 17 years and 3 months old. (b) Superimposition of pretreatment (black), posttreatment (red), and follow-up (green) cephalometric tracings on stable structures of anterior cranial base. (c) Regional superimposition of cephalometric tracings on the maxilla and mandible.

At the end of functional and full-fixed treatments, superimposition on the anterior cranial base [20] showed a notable forward displacement of the Pog and counter-clockwise rotation of the mandible (Figure 13(b)), with SN to GoGn reduced by 6.7° (Table 3). The regional mandibular superimposition [20] demonstrated upward growth of the condyle, along with extrusion of the molars and an irrelevant change in the incisor inclination (Figure 13(c); Table 3). The regional maxillary superimposition [20] demonstrated slight molar extrusion and an improvement of the incisor proclination by about 6° (Figure 13(c); Table 3).

Finally, stable results were seen at the 21-month follow-up in terms of occlusion and skeletal relationship (Figure 13; Table 3). Moreover, a further improvement of the skeletal relationship was seen during this follow-up term, as ANB angle and Wits appraisal further decreased up to 0.5° and 0.8 mm, respectively. Also, Pogonion continued to move forward as the Pog to N perp. changed from -1.4 mm to +0.1 mm (Table 3). Overall, the facial aesthetic changed noteworthily, with full correction of the convex profile.

## 3. Discussion

The present case series show that noteworthy skeletal effects can be achieved in Class II malocclusion patients with relevant occlusal and aesthetic outcomes if treated during the pubertal growth phase, as assessed though the MPM method. It has also been shown that results are stable or slightly improved after functional treatment. These results would be achieved irrespective of the appliance used.

The MPM method has a simple interpretation of the stages and can be repeated over time to closely monitor the ossification events. This is of importance considering that only 1 out of 3 patients presented at MPM stage 3, while Cases 1 and 2 presented too early to begin any functional treatment. More in detail, for Cases 1 and 2, the first MPM recording began about 1 year after the first visit. As reported herein, 3 (annual) consecutive MPM recordings may be enough to plan with reasonable accuracy the timing of intervention in individual patients (even taking into account the variable durations of the stages [10, 11, 21]). The usefulness of the MPM method would also be due to the concept that the CVM method has proved to have only a moderate diagnostic accuracy in the detection of the mandibular growth peak [21], while for the HWM method, data on diagnostic accuracy has still to be reported [2].

Herein, all the patients had a high degree of compliance as showed by the electronic monitoring [2]. This may explain the satisfactory results in terms of mandibular advancement. However, the differences in the amount and direction of mandibular growth remain to the explained. The present results demonstrate that, despite satisfactorily clinical outcomes in terms of resolution of the skeletal Class II malocclusion and aesthetic improvement, the mandibular responses may be noteworthily different among patients. Therefore, other than compliance and optimal treatment timing, other factors are likely to contribute to individual responsiveness. Among these factors may be a closed gonial angle (Co-Go-Me Angle) [22], which was shown to be associated with greater responsiveness when below 125°. In Cases 1, 2, and 3, pretreatment Co-Go-Me angles were 122.1°, 123.7°, and 119.3°, respectively. Moreover, even though at present there is still poor evidence of existence of reliable predictive features for the responsiveness to functional treatment [2], the degree of mandibular advancement might be a contributing factor as it is expected that a greater advancement is responsible for a greater induced condylar growth. However, the degree of advancement for the construction of a functional appliance is limited by the occlusion. Herein, all the cases had advancement up to an incisor edge-to-edge relationship, even though this was greater in Case 1 who showed a full-cusp Class II molar relationship before treatment. Therefore, all the cases reached a good skeletal Class I relationship with skeletal effects roughly correlated with the degree of initial dental and skeletal Class II malocclusion (i.e., greatest and lower in Cases 1 and 2, respectively).

The different directions of condylar growth may be responsible for the changes in total facial divergence. More in detail, in Case 1, both condyle and ramus had an upward and backward growth response (Figure 5(c)), while in Case 2 an upward and backward growth response was also seen, but this was mainly limited to the condyle region (Figure 9(c)). Finally, in Case 3, a mostly upward growth of the condyle was seen (Figure 13(c)). In Case 3, total facial divergence decreased by almost 5° (along with an increase in the vertical lower face), while in Cases 1 and 2, the same parameter remained stable or increased no more than 3°, respectively (Tables 1-3). Future investigations are warranted to elucidate whether more vertical growth of the condyle (as in Case 3) is preferable, as the consequent counter-clockwise rotation of the mandible, and whether reliable pretreatment parameters exist to predict such a response.

Irrespective of the modality of condylar growth, the cases presented herein showed a relevant aesthetic improvement, where functional appliances were able to reduce the excess in maxillary incisor proclination (as in Cases 1 and 2, Tables 1 and 2), while causing a negligible (Case 1) or acceptable (Case 2) proclination of the mandibular incisors. Furthermore, the soft tissue profile did not worsen significantly by the retrusion of the upper lip in any of the cases.

In spite of the wide use of functional treatment for skeletal Class II malocclusion, very little data has been reported regarding the stability of the outcomes [23–25], especially when dealing with patients treated at the pubertal growth spurt [5], irrespective of the appliance used. Herein, in Case 3, follow-up was up to 21 months, accounting for almost 4 years considering the actual end of the functional treatment phase, while Cases 1 and 2 had follow-up of 18 and 16 months, respectively. All the skeletal outcomes showed to be stable, with several improvements occurring in all the three cases (Tables 1–3). For instance, the ANB angle continued to reduce up to 0.5° and Pog to N perp. also showed some further reduction over the posttreatment term (Tables 1–3). This might be the result of a better adaptation of the soft tissues to the growth and anterior displacement of the mandible.

## 4. Conclusions

Even though the present results show merits for the MPM method, more studies with larger samples are needed to confirm the benefits in terms of growth response of functional treatment for skeletal Class II malocclusion, the timing of which is based upon this method. Given the reduced radiation exposure to the patients and the easiness of the execution, the use of the MPM method may be recommended in everyday practice.

## Figures and Tables

**Table 1:** Cephalometric data of Case 1.

Parameter	Pretreatment	Posttreatment	Follow-up
Age	12 yrs, 6 mos	13 yrs, 10 mos	15 yrs, 4 mos
SNA angle	79.1°	78.9°	79.2°
SNB angle	72.8°	76.7°	77.3°
A to Nasion perp.	-2.8 mm	-2.7 mm	-2.8 mm
Pog to Nasion perp.	-12.6 mm	-7.2 mm	-6.2
ANB angle	6.3°	2.2°	1.9°
Wits appraisal	10.5 mm	1.6 mm	2.0 mm
Palatal plane to FH	4.7°	4.3°	3.9°
SN to GoGn	28.5°	31.0°	28.3°
Co-Gn distance	104.1 mm	112.0 mm	113.7 mm
Co-Go-Me angle	122.1°	123.7°	122.1°
+1 to Palatal plane	127.5°	117.3°	118.7°
-1 to mandibular plane	98.1°	96.6°	95.4°

**Table 2:** Cephalometric data of Case 2.

Parameter	Pretreatment	Posttreatment	Follow-up
Age	11 yrs, 2 mos	12 yrs, 6 mos	13 yrs, 10 mos
SNA angle	80.7°	79.9°	80.2°
SNB angle	75.4°	76.5°	77.3°
A to Nasion perp.	-0.3 mm	-0.7 mm	-0.7 mm
Pog to Nasion perp.	-6.5 mm	-4.8 mm	-4.6 mm
ANB angle	5.3°	3.3°	2.9°
Wits appraisal	4.2 mm	-0.3 mm	0.3 mm
Palatal plane to FH	2.4°	2.1°	2.2°
SN to GoGn	30.1°	31.8°	32.3°
Co-Gn distance	99.8 mm	104.6 mm	106.3 mm
Co-Go-Me angle	123.7°	124.1°	122.1°
+1 to Palatal plane	124.5°	115.6°	116.7°
-1 to mandibular plane	99.6°	103.9°	102.6°

**Table 3:** Cephalometric data of Case 3.

Parameter	Pretreatment	Posttreatment	Follow-up
Age	13 yrs, 0 mos	15 yrs, 6 mos	17 yrs, 3 mos
SNA angle	82.7°	82.3°	82.7°
SNB angle	75.6°	78.4°	79.3°
A to Nasion perp.	0.5 mm	0.6 mm	0.9 mm
Pog to Nasion perp.	-8.6 mm	-1.4 mm	0.1 mm
ANB angle	7.1°	3.9°	3.4°
Wits appraisal	7.2 mm	4.0 mm	3.2 mm
Palatal plane to FH	-3.1°	-3.7°	-4.2°
Sn to GoGn	32.2°	26.6°	26.2°
Co-Gn distance	112.0 mm	120.4 mm	121.6 mm
Co-Go-Me angle	119.3°	116.6°	117.5°
+1 to Palatal plane	104.6°	111.7°	112.6°
-1 to mandibular plane	103.7°	103.9°	103.5°
